# 

**DOI:** 10.1017/psy.2025.16

**Published:** 2025-04-25

**Authors:** Inés M. Varas

**Affiliations:** 1 Department of Statistics, Pontificia Universidad Católica de Chile, Santiago, Chile; 2 Interdisciplinary Laboratory of Social Statistics, Pontificia Universidad Católica de Chile, Santiago, Chile

## 1 Introduction

In recent years, equating methods have evolved significantly to ensure accurate and fair score comparisons across test forms. Despite their long history, they remain essential for enabling the interchangeability of scores from different test versions.

Extensive literature examines the assumptions, applications, and practical implementations of equating methods. Notably, Kolen & Brennan (2014) and von Davier et al. (2004) emphasize score comparability through continuous approximations of discrete distributions. While early methods used linear interpolation and log-linear techniques, von Davier et al. (2004) pioneered kernel smoothing, which estimates a continuous function by weighting nearby data points, making it the first comprehensive work on kernel equating (KE).

KE follows a structured five-step process: 1. presmoothing, reducing irregularities in score distributions; 2. estimation of score probabilities, relating score distributions to statistical model parameters; 3. continuization, applying kernel methods to approximate discrete distributions continuously; 4. estimation of the equating transformation; and 5. computation of the standard error of equating, assessing transformation accuracy.

Advancements within each step have expanded equating methods to accommodate diverse shapes and variability in score distributions. Some of these innovations are detailed in González & Wiberg (2017), focusing on implementation in R with packages like SNSequate (González, 2014) and kequate (Björn et al., 2013).

After 20 years of the KE methods’ book, the authors of *Generalized Kernel Equating with Applications in R* present a broader framework for kernel-based equating. This comprehensive structure integrates various methodological advancements developed for each step of the KE process, presenting a unified approach. The book not only reflects two decades of progress in developing flexible kernel-based methods but also underscores that equating remains an open area for ongoing research. While the book primarily invites readers to explore the foundational theory underlying the steps of kernel methods within a more general framework, it also provides practical applications and illustrations using statistical software.

## 2 Summary of chapters

The book is divided into three main parts, each building progressively on the topic of equating methods and the generalization proposed. In Part I (Chapters 1 and 2) a comprehensive overview of traditional equating methods is provided, introducing the foundational concepts and setting the stage for the proposed extension to KE. The second part covers each step of KE, now presented within a unified, generalized framework (Chapters 3 to 8). In the third part, applications of the generalized KE (GKE) method using real data are shown, accompanied by R code to guide readers through practical implementation. The book concludes with an appendix detailing the specific R packages versions utilized and indicating which packages are applicable at each step of the equating process.

Chapter 1 introduces equating methods from a statistical perspective, clearly outlining the components of the associated statistical model. Following the approach in González & Wiberg (2017), the equating transformation is defined as a function mapping one score scale to another, with an added emphasis on symmetry in the transformation. The chapter thoroughly discusses the fundamental concepts and methodologies of test equating, covering assumptions and data collection designs, including the single group (SG) design, equivalent group (EG) design, counterbalanced (CB), the nonequivalent group with anchor test (NEAT) design and the nonequivalent group design with covariates (NEC). Traditional equating transformations, including those derived through kernel methods are also described.

Chapter 2 addresses the question of how KE can be generalized, building on its five fundamental steps. The authors introduce alternative models for each step, including kernel models beyond the commonly used normal model for continuization and specialized accuracy measures for evaluating the equating transformation, all within a unified framework. This unification is based on precise mathematical and statistical definitions, which are rigorously developed in the book’s second part.

Chapter 3 focuses on presmoothing methods, introducing alternatives to traditional parametric log-linear models. Notably, discrete kernel estimators are presented as non-parametric options, alongside with presmoothing methods based on mixture models, including item response theory (IRT) models and the beta4 model. The latter relates observed and true scores by assuming a four-parameter beta distribution for the true score and using a two-term approximation of the compound binomial distribution for the observed score given the true score (Lord, 1965). The chapter also explores various approaches for evaluating these methods, such as the Akaike Information Criterion, the Bayesian Information Criterion, and chi-square goodness-of-fit statistics, among others. However, criteria for selecting among presmoothing methods are not addressed.

Chapter 4 addresses the second step in KE methods: estimating score probabilities within the authors’ generalized framework. The chapter presents the design functions introduced by von Davier et al. (2004). Specific design functions are shown for univariate distributions (for the EG design) and bivariate distributions for all traditional equating designs, including the SG, CB, and NEAT designs. Additionally, the form of the design function for the NEC design is included. The chapter further explores methods for estimating score probabilities, particularly through IRT models, offering readers a comprehensive view of probabilistic estimation within this generalized framework.

The continuization step in KE methods is examined across two chapters within the generalized framework. Chapter 5 specifically focuses on transforming discrete score distributions into continuous ones through kernel methods, formally presenting this process as a convolution of density functions associated with random variables. The chapter summarizes several alternatives to the normal kernel proposed by von Davier et al. (2004) and already applied in the literature, including logistic, uniform, Epanechnikov, and adaptive kernels. Additionally, the theory of cumulants is introduced as an alternative approach for assessing the quality of the continuous approximation to the original discrete distribution.

Chapter 6 delves into the role of the bandwidth parameter in balancing the level of smoothing within the GKE framework. Several methods from kernel density estimation have been adapted to the KE context, offering alternatives to the penalty function originally described by von Davier et al. (2004). These methods include leave-one-out, likelihood cross-validation, and double smoothing approaches, among others, providing a comprehensive toolkit for achieving optimal smoothing in the equating process.

Chapter 7 addresses the equating transformation which is the fourth step in KE. This chapter presents the transformation as a flexible tool, adaptable for use with observed score equating methods, equating methods based on IRT models, and local equating approaches.

The final step in KE is evaluating the equating transformation. Within the GKE framework, evaluation metrics fall into two categories: *equating-specific measures* and *statistical evaluation measures*. The former includes metrics designed specifically for equating, such as the difference that matters, which compares equated scores and scale scores, and the standard error of equating, which measures the variability of equated scores. Statistical evaluation methods include classical measures such as bias and root mean square error. When the equating transformation is treated as the parameter of interest, these metrics can be derived from simulated score data. The chapter also provides a detailed discussion on implementing evaluation procedures using simulated data, offering guidance on ensuring accurate and meaningful assessments of the equating transformation.

Chapters 9 and 10 offer practical examples of several methods covered in the generalized framework proposed, applying them to the EG and NEAT designs using real datasets as also discussed in González & Wiberg (2017). For the EG design, the analysis relies mainly on the SNSequate R package (González, 2014), while the NEAT design is demonstrated through the kequate R package (Björn et al., 2013). These chapters provide readers with hands-on guidance for implementing the GKE framework, effectively connecting the theory explored in the book with real-world examples and accessible R code.

## 3 Conclusion

The book offers a well-structured exploration of approaches alternatives to those described in von Davier et al. (2004) on each of the steps of KE to define a general framework. In authors’ words *...(it) stipulates that we will have several methods that we can choose between in each of the five steps that are used to conduct an equating*. A strength of the book is its flexibility, allowing readers to design their own equating process by selecting among various options for each step. While the authors do not advocate for a specific approach, they provide a thorough discussion of the assumptions underlying each option and offer guidance on evaluating the choices made at each stage.

The book presents several results that mark significant advancements within the generalized framework proposed. Notably, in contrast to traditional equating approaches, GKE defines the equating transformation as a symmetric transformation. This definition formalizes the symmetry assumption as the only one directly related to the properties of the equating function itself, reflecting recent discussions of the symmetry property in equating (van der Linden, 2019). In a similar context, in the NEAT design, score distributions conditional on anchor scores are introduced as identification restrictions, enhancing the relevance of the definition of the statistical model under the different equating designs. Additionally, the formalization of kernel methods as a convolution of distribution functions enables a comprehensive analytical evaluation of KE techniques. The inclusion of a wide range of statistical tools for in-depth assessment of the equating function through classical statistical evaluation further underscores how equating methods can benefit from advancements in statistical analysis.

By defining the equating problem from a statistical modeling perspective, as outlined in González & Wiberg (2017), the authors effectively show how essential concepts-such as model identification, parameter estimation, and the evaluation of estimates-play a central role in equating methods. One of the book’s major strengths is its integration of new models for the presmoothing step, methods for estimating score distributions in several settings, alternative kernels in the continuization step, and various metrics for assessing the equating transformation, all within a unified framework. This achievement is grounded by the authors’ rigorous statistical and mathematical formalization of each step in the KE process.

The book maintains a clear progression, with each chapter introduced concisely and concluded with a structured summary, ensuring a coherent framework. However, frequent references to upcoming chapters occasionally disrupt the reading flow.

In line with the authors’ intended audience, I highly recommend this book to graduate students, test developers, and psychometricians with background in statistical modeling, estimation, and inference. With practical method illustrations, only basic R knowledge is needed for GKE implementation. Statisticians will also find the book a valuable resource. Beyond its applications, the book offers valuable insights into advanced statistical methodologies in psychometrics and related fields.
